# Higher premature atrial contraction burden after radiofrequency ablation vs. pulsed field or cryoballoon ablation in paroxysmal atrial fibrillation: a 3-year follow-up retrospective study

**DOI:** 10.3389/fcvm.2025.1627579

**Published:** 2025-09-12

**Authors:** Yongxing Jiang, Chenxu Luo, Mingjun Feng, Yibo Yu, Xianfeng Du, Caijie Shen, Guohua Fu, Binhao Wang, Renyuan Fang, He Jin, Fang Gao, Huimin Chu

**Affiliations:** Cardiac Arrhythmia Center, The First Affiliated Hospital of Ningbo University, Ningbo, China

**Keywords:** premature atrial contraction, paroxysmal atrial fibrillation, pulsed field ablation, cryoballoon ablation, radiofrequency ablation

## Abstract

**Background:**

Pulsed field ablation (PFA), a novel non-thermal energy source, has shown favorable 1-year data on the efficacy and safety profile in the treatment of paroxysmal atrial fibrillation (PAF). We sought to compare PFA, cryoballoon ablation (CBA), and radiofrequency ablation (RFA) in PAF treatment in a 3-year follow-up period.

**Methods:**

Patients with PAF undergoing first-time catheter ablation by PFA, CBA, and RFA were retrospectively included. The procedure endpoint was pulmonary vein isolation (PVI). Patients were followed with 24 h ambulatory ECG monitoring at 1, 3, 6, and 12 months and every 6 months thereafter. The primary efficacy endpoint was freedom from any atrial tachyarrhythmia >30 s occurring after the 3-month blanking period.

**Results:**

A total of 280 PAF patients undergoing ablation with PFA (*n* = 65), CBA (*n* = 55), or RFA (*n* = 160) were enrolled. The mean age was 60.9 ± 8.7 years, with 55.7% male patients (*n* = 156). Acute PVI was achieved in all patients. Total procedural time was shortest with PFA [91.0 (85.0, 103.0) min, *P* < 0.001], whereas fluoroscopy time was shortest with RFA [9.0 (7.0, 10.0) min, *P* < 0.001]. The peri-procedural complication rate was 2.5%. The Kaplan–Meier estimated 3-year freedom from any atrial tachyarrhythmia >30 s was 76.9% with PFA, 72.7% with CBA, and 66.9% with RFA (log-rank *P* = 0.298). The principal finding of the study was the significantly lowest premature atrial contraction (PAC) burden in non-recurrent patients treated with PFA (0.04%) compared with CBA (0.05%) and RFA (0.11%) (*P* < 0.001).

**Conclusion:**

At the 3-year follow-up, arrhythmia freedom was similar in PFA, CBA, and RFA in patients with PAF. PFA and CBA contributed to significantly lower PAC burden compared with RFA in patients without recurrence.

## Introduction

Catheter ablation as first-line therapy for atrial fibrillation (AF) not only improves quality of life but also decreases the rates of stroke and mortality (1). Standard thermal ablation, such as point-by-point radiofrequency ablation (RFA) and cryoballoon ablation (CBA), is routinely performed in catheter ablation of paroxysmal AF (PAF), relying on time-dependent conductive heating or cooling (2, 3). Despite tremendous advances in thermal ablation methods, it is still challenging to maintain durable pulmonary vein isolation (PVI) and avoid collateral damage of surrounding tissue (4, 5). Pulsed field ablation (PFA) is a novel non-thermal ablation modality, applying ultrarapid electrical pulses to destabilize cell membranes and culminating in cell death (6). A large number of studies demonstrated that PFA was effective in treating AF with excellent tissue-selective modality (7–9).

Recently, studies comparing PFA, CBA, and RFA in the treatment of PAF demonstrated that PFA was as good as RFA and CBA at 1-year follow-up (10, 11). However, there is no literature directly comparing the long-term outcomes of different energy sources in catheter ablation of PAF. In this trial, we aimed to compare the efficacy and safety of PFA, CBA, and RFA for PAF treatment in a 3-year follow-up period.

## Methods

### Trial design

This trial was a retrospective, observational, single-center study conducted at The First Affiliated Hospital of Ningbo University, China. The trial retrospectively included patients undergoing the first-time catheter ablation of non-valvular PAF by PFA, CBA, and RFA between January 2021 and March 2022. The PFA cohort was the participants enrolled in the PLEASE-AF study (12). The trial was approved by the Institutional Review Board of The First Affiliated Hospital of Ningbo University, in compliance with the principles of the Declaration of Helsinki. All participants gave their written informed consent. The study protocol was approved by the ethics committee of The First Affiliated Hospital of Ningbo University.

### Study population

Symptomatic PAF patients (18–75 years of age), who were treated with at least one antiarrhythmic drug (AAD) that was ineffective or intolerable, were enrolled. PAF was defined, according to the 2023 ACC/AHA/ACCP/HRS guidelines for the diagnosis and management of AF, as AF that is intermittent and terminates within 7 days of onset (2). The trial excluded patients with non-paroxysmal AF, AF secondary to reversible or non-cardiac causes, valvular AF, hypertrophic cardiomyopathy, atrial thrombosis, and left atrial diameter of >55 mm. Patients were included in the final analysis only if they had undergone ablation ≥3 years earlier and had documented follow-up data at both 12-month and 36-month timepoints (with 30-month follow-up permitted as an acceptable alternative). All participants gave written informed consent for the ablation procedure and data collection.

### Ablation procedure

All ablation procedures were performed under general anesthesia, with uninterrupted oral anticoagulation and intravenous heparin to maintain the activated clotting time between 300 and 350 s. Transesophageal echocardiography was performed in all patients to exclude intracardiac thrombi on the day of the ablation procedure. AAD therapy, except amiodarone, was discontinued at least five half-lives before the ablation procedure. After femoral venous access was obtained, the coronary sinus and ventricular electrodes were routinely placed.

### Pulse field ablation procedure

The CardiPulse™ PFA System (Hangzhou Dinova EP Technology Co., Ltd, China) has been previously described elsewhere (12). This consists of an 11 F hexaspline PFA catheter, a 12 F steerable sheath, and a portable touch screen pulsed field generator. After a single transeptal puncture, the PFA catheter assisted by the deflectable sheath was advanced into the LA using a guidewire. Electroanatomic mapping and fluoroscopy were used to guide PFA catheter positioning at the pulmonary vein (PV) ostium, and baseline electrical potentials were recorded from all PVs. Typically, PV ablation commenced at the left superior pulmonary vein (LSPV), followed by the left inferior pulmonary vein (LIPV), right superior pulmonary vein (RSPV), and right inferior pulmonary vein (RIPV). The PFA catheter was adjusted to a “basket” configuration for PV ostium ablation and then switched to a “flower” pose for PV antrum ablation. The PFA catheter could adjust to a maximal diameter of 28, 32, or 36 mm in the “flower” configuration. All PFA applications were delivered in a biphasic–bipolar waveform with different pulse intensities at the PV ostium (1,800 V) and PV antrum (2,000 V). Generally, two to three ostial sites and three antral sites were required to achieve full circumferential isolation of PVs. The duration of a single application was 2 s.

### Cryoballoon ablation procedure

The cryoballoon ablation protocol has been clearly described in previous articles (13, 14). A fourth-generation cryoballoon (ArtFreezer™, ArtechMed, Shanghai, China) was inserted with the use of a transseptal puncture and an over-the-wire delivery technique. A 23, 28, or 32 mm cryoballoon was placed at each PV until it was occluded. Two cyroballoon applications, the first 180 s in duration and an additional 120 s, were recommended for each pulmonary vein. Following PVI, the additional cryoapplication was delivered after the rewarming phase (to +32 °C). If PVI was not achieved or maintained, bonus applications were permitted. Continuous phrenic nerve pacing with intervals of 1,000 ms was required during cryoballoon ablation of the right-sided PVs.

### Radiofrequency ablation procedure

A high-power short-duration RF ablation protocol has been described in previous publications (15, 16). Two transseptal punctures were performed via a fixed sheath (SL1, Abbott, Saint Paul, MN, USA) or a steerable sheath (Vizigo, Biosense Webster, CA, USA) at the operator's discretion. A 3D electroanatomical mapping system (CARTO 3, Biosense Webster, CA, USA) was used to guide mapping and ablation. Left atrial electroanatomical mapping was achieved via a multipolar catheter (PentaRay, Biosense Webster, CA, USA), and RF ablation was performed with a contact force-sensing, open-irrigated, radiofrequency catheter (THERMOCOOL SMARTTOUCH Surround Flow, Biosense Webster, CA, USA). Circumferential PVI was performed with RF delivered in a point-by-point manner, circumferentially around each ipsilateral set of PVs with a power of 45 W, a desired contact force of 10–15 g, and a target ablation index of 500 for anterior and 400 for posterior PV segments. Non-pulmonary vein ablation, including mitral isthmus, tricuspid isthmus, and LA roof linear ablation and superior vena cava isolation, was not recommended.

For all the ablation methods, if the sinus rhythm was not restored, direct current cardioversion was performed. The procedure endpoint was PVI confirmed by entrance and exit block after a 20 min observation period. Adenosine bolus injections were used to assess for acute PV reconnection and demask dormant conduction.

### Post-procedural management and follow-up

Following the ablation procedure, patients were discharged after at least one overnight observation, if no peri-procedural complications were observed. Oral anticoagulation was maintained for at least 2 months, and AADs were resumed during the blanking period (3 months post-ablation) and discontinued if no arrhythmic recurrences occurred.

Follow-up visits were scheduled at the 1, 3, 6, and 12 months and every 6 months thereafter, including physical examination, 12-lead ECG, and 24 h ambulatory ECG monitoring. If symptoms of arrhythmia recurrence occurred, additional 24 h ambulatory ECG monitoring was prescribed. Arrhythmia recurrence patterns and timing data were also recorded for all patients with AF recurrence. Premature atrial contraction (PAC) was defined as supraventricular complexes occurring >30% earlier than expected compared with the previous R–R interval. The numbers of single, paired, and short-run PACs were quantified in 24 h ambulatory ECG monitoring.

### Study endpoints

The primary efficacy endpoint was freedom from any atrial tachyarrhythmia >30 s occurring after the 3-month blanking period, the use of class I or III AADs or cardioversion after the 3-month blanking period, or redo procedures. The primary safety endpoint was any procedure-related serious adverse events, including groin hematoma, pseudoaneurysm, pericardial effusion, cardiac tamponade, symptomatic pulmonary vein stenosis, unresolved phrenic nerve injury, transient ischemic attack, stroke, myocardial infarction, major bleeding, atrioesophageal fistula, and death.

### Statistical analysis

Continuous data were reported as mean ± standard deviation or median [interquartile range] and compared using the Kruskal–Wallis test or ANOVA as appropriate; categorical data were reported as number (percentages) and compared using Pearson's chi-square test or Fisher’s exact test. The D’Agostino and Pearson normality test was used to assess normality. Freedom from atrial tachyarrhythmia recurrence was analyzed using the Kaplan–Meier method. Univariate and multivariate Cox proportional hazard models were used to evaluate predictors of AF recurrences. The proportional hazards assumption for all covariates in the Cox regression models was tested by Schoenfeld residual tests. All tests were two-sided, and a two-tailed *P*-value less than 0.05 was deemed statistically significant. All statistical analyses were performed using SPSS 27 (SPSS, Chicago, IL, USA) and GraphPad Prism 7.0 software (GraphPad Software Inc., San Diego, CA, USA).

## Results

### Patients characteristics

A total of 280 PAF patients who received first-time catheter ablation with either PFA (*n* = 65), CBA (*n* = 55), or RFA (*n* = 160) were enrolled in analysis (Supplementary Figure S1). Baseline characteristics of the enrolled patients are presented in Table 1. The mean age of enrolled patients was 60.9 ± 8.7 years, with 55.7% male patients (*n* = 156), and hypertension was the most common comorbidity (53.6%). The demographic characteristics, medical history, and echocardiogram parameters were well matched among groups.

**Table 1:** Baseline characteristics.

Characteristic	PFA (*n* = 65)	CBA (*n* = 55)	RFA (*n* = 160)	*P*-value
Age, years	61 [56, 67]	62 [56, 67]	62 [55, 68]	0.959
Female, *n* (%)	29 (44.6)	22 (40.0)	73 (45.6)	0.529
BMI, kg/m^2^	23.9 [22.2, 25.8]	24.2 [22.0, 26.2]	23.4 [21.5, 25.3]	0.245
Hypertension, *n* (%)	41 (63.1)	29 (52.7)	80 (50)	0.202
Diabetes, *n* (%)	9 (13.8)	5 (9.1)	16 (10)	0.637
Heart failure, *n* (%)	1 (1.5)	1 (1.8)	5 (3.1)	0.879
Coronary artery disease, *n* (%)	6 (9.2)	6 (10.9)	13 (8.1)	0.400
Stroke, *n* (%)	3 (4.6)	1 (1.8)	10 (6.3)	0.487
Left atrial diameter, mm	35.0 [32.0, 37.5]	36.0 [34.0, 39.0]	35.0 [33.0, 39.0]	0.310
LVEF, %	65.0 [61.0, 69.0]	67.0 [63.0, 69.0]	66.0 [62.0, 70.0]	0.692
CHA_2_DS_2_-VASC, score	2 [1, 3]	2 [1, 3]	2 [1, 3]	0.451

Continuous data were reported as mean ± standard deviation or median [interquartile range], and categorical data were reported as number (percentages).

BMI, body mass index; LVEF, left ventricular ejection fraction; PFA, pulsed field ablation; CBA, cryoballoon ablation; RFA, radiofrequency ablation.

### Procedural characteristics

A total of 1,088 PVs were ablated, and acute PVI was achieved in all of the enrolled patients.

In the PFA group, 267 PVs were ablated, and the mean number of applications per patient was 25.2 ± 2.3. A 32 mm PFA catheter was the device of choice in the majority of cases (*n* = 64, 98.5%), and a 36 mm PFA catheter was used in only one patient.

In the CBA group, 176 PVs were ablated. The mean number and time of applications per patient were 9.5 ± 1.9 and 1,350.0 ± 280.6 s, respectively. A 28 mm cryoballoon was selected in all patients except one selected a 32 mm cryoballoon.

In the RFA group, 646 PVs were ablated. Additional ablations beyond the pulmonary veins were not performed.

Procedural details are shown in Table 2. The total procedural time was shortest with PFA [91.0 (85.0, 103.0) min, *P* < 0.001], whereas the fluoroscopy time was shortest with RFA [9.0 (7.0, 10.0) min, *P* < 0.001; Table 2].

**Table 2:** Procedure characteristics and complications.

Characteristic	PFA (*n* = 65)	CBA (*n* = 55)	RFA (*n* = 160)	*P*-value
Total procedural time, min	91.0 [85.0, 103.0]	101.0 [90.0, 115.0]	107.0 [94.0,123.8]	<0.001
Fluoroscopy time, min	19.0 [16.0, 22.0]	19.0 [16.0, 26.0]	9.0 [7.0, 10.0]	<0.001
Acute PVI, *n* (%)	65 (100)	55 (100)	160 (100)	>0.999
Number of PV	267	176	646	
Total complications, *n* (%)	3 (4.6)	1 (1.8)	3 (1.9)	0.321
Groin hematoma, *n* (%)	1 (1.5)	0 (0)	2 (1.3)	
Bleeding, *n* (%)	0 (0)	1 (1.8)	0 (0)	
Pericardial effusion	0 (0)	0 (0)	1	
Cardiac tamponade, *n* (%)	0 (0)	0 (0)	0 (0)	
Renal function Impairment, *n* (%)	2 (3.1)	0 (0)	0 (0)	
Stroke, *n* (%)	0 (0)	0 (0)	0 (0)	
AE fistula, *n* (%)	0 (0)	0 (0)	0 (0)	
Death, *n* (%)	0 (0)	0 (0)	0 (0)	

Continuous data were reported as mean ± standard deviation or median [interquartile range], and categorical data were reported as number (percentages).

PVI, pulmonary vein isolation; PV, pulmonary vein; AE fistula, atrioesophageal fistula; PFA, pulsed field ablation; CBA, cryoballoon ablation; RFA, radiofrequency ablation.

### Efficacy outcomes

Within the 3-month blanking period, the class I or III AAD therapy was used in 55 (84.6%) PFA, 46 (83.6%) CBA, and 138 (86.2%) RFA patients. Three patients in the PFA group, three patients in the CBA group, and eight patients in the RFA group were still using the class I or III AAD after the blanking period.

**Figure 1:**
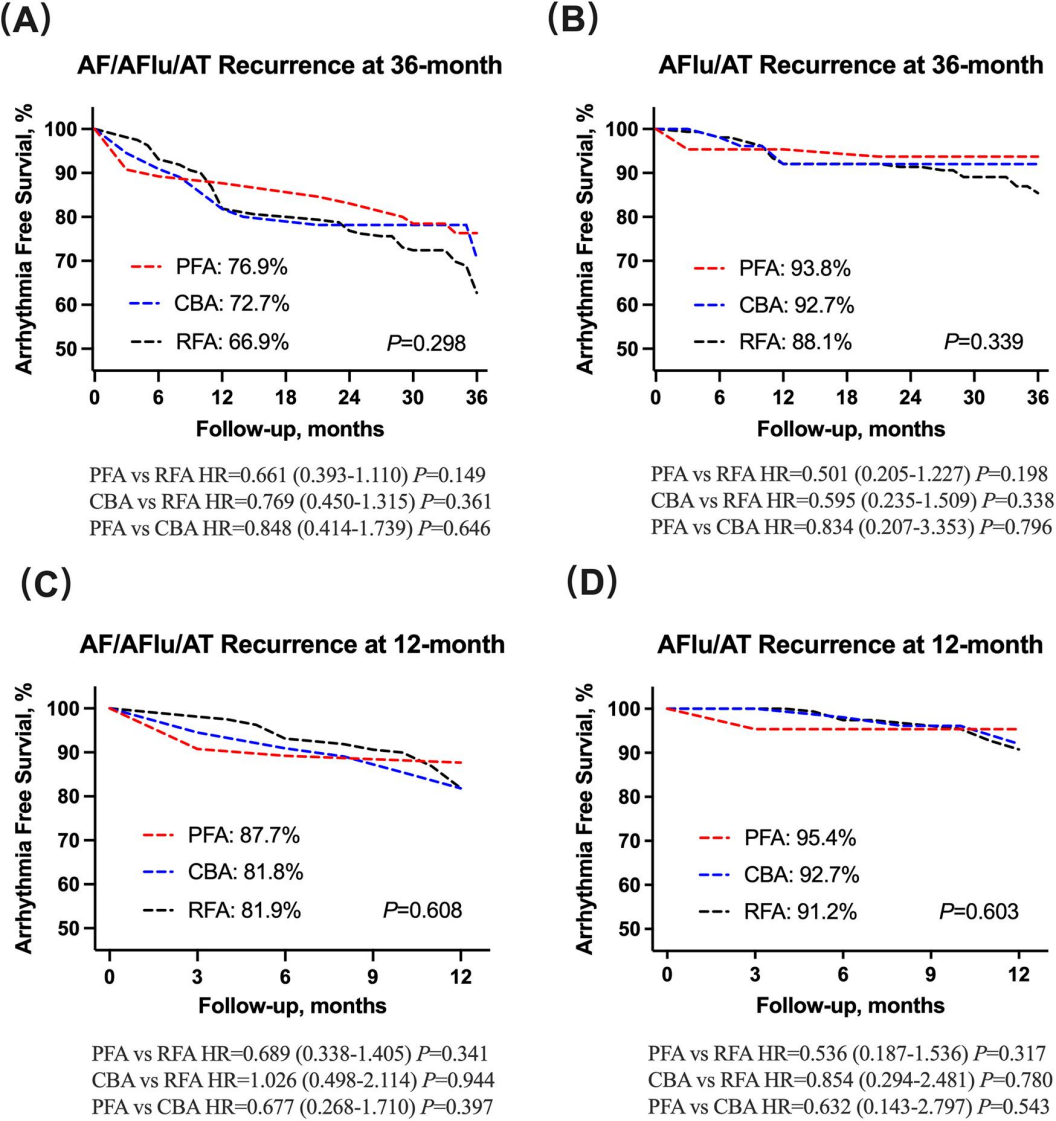
**(A,B)** AF/AFlu/AT and AFlu/AT recurrence at 36-month follow-up has no statistical difference among PFA, CBA and RFA groups. **(C,D)** AF/AFlu/AT and AFlu/AT recurrence at 12-month follow-up has no statistical difference among PFA, CBA, and RFA groups. AF, atrial fibrillation; AFlu, atrial flutter; AT, atrial tachycardia; PFA, pulsed field ablation; CBA, cryoballoon ablation; RFA, radiofrequency ablation.

The median follow-up duration in patients without arrhythmia recurrence was 35.0 (32.0, 36.0) months. Overall, 83 (29.6%) patients had atrial arrhythmia recurrence, of whom 56 were with AF and 27 were with atrial flutter or atrial tachycardia (Table 3). The long-term Kaplan–Meier estimated freedom from any atrial tachyarrhythmia >30 s was 76.9% with PFA, 72.7% with CBA, and 66.9% with RFA (log-rank *P* = 0.298; Figure 1A). There were no statistical differences in atrial flutter or atrial tachycardia recurrence among the PFA, CBA, and RFA groups (log-rank *P* = 0.339; Figure 1B). Baseline and procedural variables were included for the univariate and multivariate Cox model for assessing the role of atrial tachyarrhythmia recurrence after the index procedure (Supplementary Table S1). The proportional hazards assumption in the Cox model was met for all covariates (all Schoenfeld test *P* > 0.05).

**Table 3:** Follow-up characteristics.

Characteristic	PFA (*n* = 65)	CBA (*n* = 55)	RFA (*n* = 160)	*P*-value
Long-term follow-up				
AF/AFlu/AT, *n* (%)	15 (23.1)	15 (27.3)	53 (33.1)	0.298
AFlu/AT, *n* (%)	4 (6.2)	4 (7.3)	19 (11.9)	0.339
1-year follow-up				
AF/AFlu/AT, *n* (%)	8 (12.3)	10 (18.2)	29 (18.1)	0.608
AFlu/AT, *n* (%)	3 (4.6)	4 (7.3)	14 (8.75)	0.603
Follow-up duration, months	36.0 [33.0, 36.0]	36.0 [35.0, 36.0]	35.0 [32.0, 36.0]	0.053
Second-time ablation, n	7	6	20	0.547
Durable PVI, *n* (%)	4 (57.1)	3 (50)	7 (35)	
PV reconnection, *n* (%)	3 (42.9)	3 (50)	13 (65)	

Continuous data were reported as mean ± standard deviation or median [interquartile range], and categorical data were reported as number (percentages).

AF, atrial fibrillation; AFlu, atrial flutter; AT, atrial tachycardia; PVI, pulmonary vein isolation; PV, pulmonary vein; PFA, pulsed field ablation; CBA, cryoballoon ablation; RFA, radiofrequency ablation.

At 1-year follow-up, a total of 47 (18.3%) patients had arrhythmia relapse (Table 3). The 1-year treatment success was 87.7% for PFA, 81.8% for CBA, and 81.9% for RFA, estimated by Kaplan–Meier analysis (log-rank *P* = 0.608; Figure 1C). In addition, no statistical differences were observed in atrial flutter or atrial tachycardia recurrence among the PFA, CBA, and RFA groups (log-rank *P* = 0.603; Figure 1D).

There were 7 of PFA, 6 of CBA, and 20 of RFA patients who received a second-time ablation. PV reconnection was detected in 42.9% PFA (*n* = 3), 50% CBA (*n* = 3), and 65% RFA (*n* = 13) patients (*P* = 0.547, Table 3).

**Figure 2:**
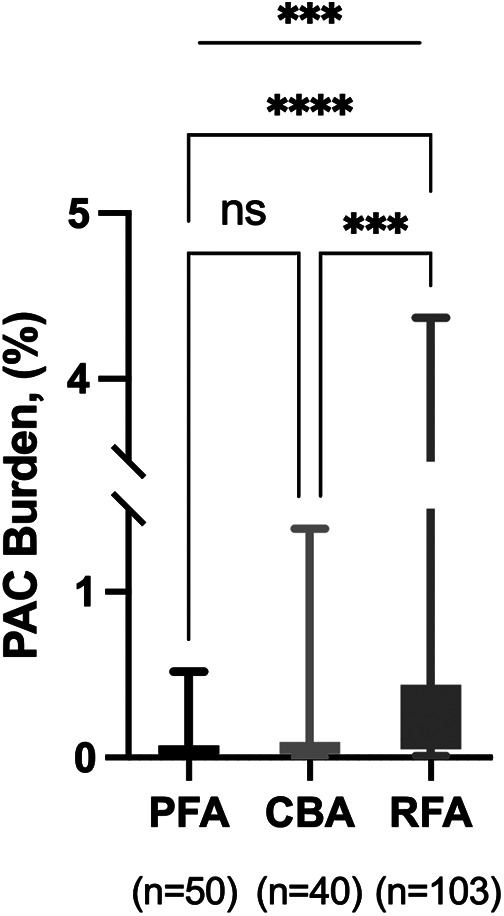
PAC burden in patients without recurrence. PFA, pulsed field ablation; CBA, cryoballoon ablation; RFA, radiofrequency ablation; PAC, premature atrial contraction. ***P < 0.001; ****P < 0.0001.

In patients without arrhythmia recurrence, PAC burden at the last time of 24 h ambulatory ECG monitoring was highest with RFA, compared with PFA and CBA (0.11 [0.05, 0.44]% vs. 0.04 [0.02, 0.07]% vs. 0.05 [0.02, 0.10]%, *P* < 0.001; Table 4, Figure 2). The median number of PAC beats was 107 [49.0, 452.0] with RFA, 38.0 [23.0, 74.0] with PFA, and 54.5 [19.8, 97.5] with CBA (*P* < 0.001, Table 4). Total heartbeats and heart rate were reported in Table 4, and there was no statistical difference among groups. The use of β-receptor blocker was 20% with PFA, 22.5% with CBA, and 24.3% with RFA (*P* = 0.835).

**Table 4:** 24 h Holter monitoring data in patients without recurrence.

Characteristic	PFA (*n* = 50)	CBA (*n* = 40)	RFA (*n* = 107)	*P*-value
Monitoring duration, h	23.4 [23.0, 24.0]	23.5 [23.3, 24.0]	23.5 [22.8, 24.0]	0.567
Total heartbeats	102,978 [99,427, 108,439]	102,352 [96,924, 107,031]	102,355 [96,323, 106,377]	0.450
Heart rate, beats/min				
Maximum	118.3 ± 8.5	115.9 ± 8.5	115.1 ± 9.4	0.124
Minimum	49.5 ± 6.1	49.2 ± 6.1	47.4 ± 6.3	0.090
Mean	74.0 [71.0, 76.3]	72.5 [69.0, 75.8]	72.0 [70.0, 75.0]	0.128
PAC, beats	38.0 [23.0, 74.0]	54.5 [19.8, 97.5]	107.0 [49.0, 452.0]	<0.001
PAC burden, %	0.04 [0.02, 0.07]	0.05 [0.02, 0.10]	0.11 [0.05, 0.44]	<0.001

Continuous data were reported as mean ± standard deviation or median [interquartile range], and categorical data were reported as number (percentages).

PAC, premature atrial contraction; PFA, pulsed field ablation; CBA, cryoballoon ablation; RFA, radiofrequency ablation.

### Safety outcomes

A total of seven peri-procedural adverse events were reported during the follow-up period. One patient in the PFA group and one patient in the RFA group had a groin hematoma, which was managed conservatively without intervention. One patient in the CBA group experienced acute upper gastrointestinal bleeding on the day of ablation, which was treated with medicine. One patient in the RFA group developed a pericardial effusion on the day of ablation, which was resolved with percutaneous drainage. Another patient in the RFA group developed a pseudoaneurysm, which was managed surgically.

Two patients in the PFA group experienced acute kidney injury. One patient with baseline renal dysfunction (serum creatinine 110 µmol/L) had a post-ablation increase in serum creatinine to 146 umol/L, and indirect bilirubin increased from 8.15 to 21.4 µmol/L supporting hemolysis. The other patient's serum creatinine increased from 77 to 111 µmol/L, with concomitant indirect bilirubin increased from 15.3 to 23.8 µmol/L. Both patients recovered to the baseline level of kidney function after receiving fluid infusion therapy.

There were no additional adverse events, either intraprocedural or during follow-up, including transient ischemic attack, stroke, PV stenosis, phrenic nerve injury, atriooesophageal fistula, or death.

## Discussion

Herein, we described the 3-year results of different energy sources in catheter ablation of PAF in a single-center, observational study. The main findings of this study were as follows. (i) PAC burden in patients without arrhythmia recurrence was higher in RFA, compared with PFA and CBA. (ii) Long-term freedom from atrial arrhythmia was high and not statistically different among PFA, CBA, and RFA technologies. (iii) Acute PVI was achieved in all PVs. PFA was most effective in total procedural time, and RFA took the shortest time in x-ray exposure. (iv) All ablation strategies showed an excellent safety profile, and total procedural-related adverse events were rare.

### Efficacy

Despite tremendous advances in drug therapy and catheter ablation, the optimal treatment strategy for AF remains undetermined. The CABANA trial significantly elevated the status of catheter ablation in AF treatment by demonstrating its efficacy in markedly reducing AF recurrence and improving quality of life, though it failed to show a statistical reduction in the primary composite endpoint (17).

Thermal ablation, including RFA and CBA, is the dominant energy source for AF ablation. The FIRE AND ICE trial demonstrated comparable overall efficacy and safety profiles between the two approaches (3). PFA with high tissue-selective modality expands the landscape of ablation energy selection (6). A series of PFA studies have demonstrated its efficacy in PAF treatment in a 1-year follow-up (7, 9, 18). In the combined IMPULSE, PEFCAT, and PEFCAT II trials, the first-in-human use of PFA for PAF treatment, the Kaplan–Meier estimated 1-year freedom from atrial arrhythmia was 84.5% (9). In the MANIFEST-PF trial, a large retrospective multinational post-approval clinical use of PFA study, the 1-year freedom from atrial arrhythmia was 81.6% in the PAF cohort (7). The ADVENT trial, the only published randomized pivotal trial, further demonstrated that PFA was non-inferior to conventional thermal ablation, with respect to clinical success and safety profile at 1-year follow-up (18).

There are few data directly compared PFA, CBA, and RFA in PAF treatment. Recently, Della Rocca et al. (11) reported that the 1-year follow-up data showed a similar arrhythmia freedom (79.3% with PFA, 74.7% with CBA, and 72.4% with RFA) and a higher rate of PV reconnection in post-CBA and post-RFA redo procedures. In another propensity score-matched analysis of patients with PAF, Maurhofer et al. (10) reported that freedom from atrial arrhythmia at 1 year after PVI using PFA was as good as for PVI with CBA or RFA (85.0% with PFA, 66.2% with CBA, and 73.8% with RFA). In our study, the 1-year treatment success was 80.6% with PFA, 83.9% with CBA, and 80.6% with RFA. These results were in line with the above-mentioned findings.

To our knowledge, there is no literature that compared different energy sources in catheter ablation of PAF in such a long follow-up period. In the median 35-month follow-up period, freedom from atrial arrhythmia was achieved in 76.9% of PFA, 72.7% of CBA, and 66.9% of RFA. It is noteworthy that the result was in favor of PFA but did not achieve statistical significance, which may be due to the small sample size. These favorable results may be explained by PFA’s advantages over traditional thermal ablation methods, which had a short blanking period and had a lower rate of PV reconnected compared with CBA and RFA verified in redo procedures (11, 19). PV reconnection rates in protocol-based and planned remapping studies were reported in a recent meta-analysis: reconnection rates of at least one PV (per patient analysis) were 54% for RFA, 46% for CBA, and 30% for PFA, while a per-PV analysis revealed reconnection rates of 29% for RFA, 21% for CBA, and 13% for PFA (20). However, this opinion is still debatable. In a prospective study enrolled patients undergoing repeat ablation following index PFA or CBA for AF, electrical PV reconduction rates and patterns were similar (16/22, 73% for PFA vs. 33/44,75% for CBA) (21).

The principal finding of our study is that the PAC burden was higher in RFA compared with PFA and CBA in patients without recurrence. The mean PAC burden was 0.41 ± 0.70%, and the mean number of PAC was 420.3 ± 717.8 beats in the RFA group. However, the clinical relevance of this high PAC burden in the RFA group remains uncertain. Many studies have shown an association between PAC and new-onset AF. Prasitlumkum et al. (22) reported frequent PAC associated with up to threefold increased risk of new-onset AF and suggested that frequent PAC in the general population was an independent predictor of new-onset AF. Cabrera et al. (23) reported that the PAC burden >0.2% was an independent predictor for new-onset AF. Moreover, frequent PAC was associated with late recurrence of AF (24, 25). The mechanism of high PAC burden associated with AF late recurrence may result from reconnection in the isolation lines surrounding the PV. In fact, PV reconnection is reported to be the most frequent electrophysiological mechanism of recurrent AF after point-by-point ablation mode as RFA (25).

The current study cannot determine the underlying mechanism by which PFA and CBA reduce PAC burden compared with RFA in the long-term follow-up. Current evidence from protocol pre-specified invasive PV reassessment voltage mapping studies is largely limited to short- and mid-term follow-up data. Kawamura et al. (26) reported the voltage mapping results at 75 days after the index procedure and demonstrated catheter-based PVI with the pentaspline PFA catheter created chronic PV antral isolation areas encompassing thermal energy ablation. However, the electrophysiological profile diverges significantly in the setting of AF recurrence. Della Rocca et al. (11) reported that PFA patients showed a significantly lower number of reconnected PVs compared with those initially treated with CBA or RFA at the time of repeat ablation. AF is one of the most prevalent cardiac arrhythmias, for which the optimal treatment strategy remains undetermined (27–29). The pathogenesis of AF involves complex interactions with atrial cardiomyopathy, gut microbiome composition, and mitochondrial dysfunction, while the optimal pharmacological and device-based therapeutic strategies remain controversial (30–34). Further studies should compare the long-term voltage mapping results among the three ablation methods.

### Safety

The safety profiles of PFA, CBA, and RFA were all well-acceptable in our study. These findings are consistent with the safety observed in other literature on catheter ablation of AF (13, 35, 36). No energy-related complications, including esophageal damage, persistent phrenic injury, PV stenosis, or coronary spasm, were observed in the whole cohort.

Notably, hemolysis was observed, and two patients in the PFA group experienced acute renal injury. High-voltage pulses can cause hemolysis. Hemolysis of red blood cells causes the release of hemoglobin and can trigger the tubular barrier deregulation and oxidative cell damage, resulting in acute kidney injury (37). There are few data available regarding the occurrence of hemolysis and the subsequent impact on renal function after PFA (38). A positive correlation [*r* = 0.62 (95% CI, 0.33–0.80); *P* < 0.001] between hemolysis biomarkers and the number of PFA deliveries was identified in a multicenter analysis (39). The conclusion of this analysis that hemolysis is a frequent finding after PFA is in accordance with our findings. The clinical impact of PFA-associated hemolysis, especially on renal function impairment, is not fully investigated. In the retrospective MANIFEST-17K study, which assessed the safety of PFA in a very large cohort of >17,000 patients, hemolysis-related acute renal failure necessitating hemodialysis occurred in 0.03% of patients (5 of 17,642) (40). Planned fluid infusion immediately after the PFA procedure could prevent the renal insult (38).

### Limitations

Our findings have to be interpreted in the light of several limitations. First, our study is limited by its retrospective design and small sample size, which inherently possess limitations and biases. The PFA cohort was drawn from the PLEASE-AF study, which may have potential variations in protocol standardization. Our study relied exclusively on historical data collection; prospective randomized controlled trials are warranted to validate the findings of this study.

Second, the monitoring strategy, a 24 h ambulatory ECG monitoring, used to assess atrial arrhythmia recurrence might underestimate the results of recurrences. However, intensive frequency of follow-up visits was scheduled at 1, 3, 6, and 12 months and every 6 months thereafter. Third, the cohort of our study only enrolled patients with paroxysmal AF; the findings cannot be extended to patients with non-paroxysmal AF. Fourth, the finally analyzed patients mandating complete 12- and 36-month follow-up likely created a selection bias by excluding less adherent patients. Fifth, the study only observed the clinical outcome of a higher PAC burden after RFA, the underlying mechanism was not fully explored, and the clinical relevance of PAC burden was not evaluated by any symptom questionnaire.

## Conclusions

In the 3-year follow-up, arrhythmia freedom was similar in PFA, CBA, and RFA in patients with PAF. PFA and CBA contributed to significantly lower PAC burden compared with RFA in patients without recurrence.

## Data Availability

The raw data supporting the conclusions of this article will be made available by the authors, without undue reservation.
