# Supplementary material for: Higher premature atrial contraction burden after radiofrequency ablation vs. pulsed field or cryoballoon ablation in paroxysmal atrial fibrillation: a 3-year follow-up retrospective study
Source: Front Cardiovasc Med. 2025 Sep 12;12:1627579. doi: 10.3389/fcvm.2025.1627579 (PMC12463900; doi:10.3389/fcvm.2025.1627579)
Supplement: Supplementary file 1 [file Datasheet1.docx]

**Higher Premature Atrial Contraction Burden after Radiofrequency Ablation versus Pulsed Field or Cryoballoon Ablation in Paroxysmal Atrial Fibrillation: A Three-year Follow-up Retrospective Study**

**Supplemental Material**

Table S1. Univariate and Multivariate Cox Regression Analyses for Atrial Tachyarrhythmias Recurrence.

|  | Univariate analysis | |  | Multivariate analysis | |  |
| --- | --- | --- | --- | --- | --- | --- |
|  | HR (95%CI) | *P* value |  | HR (95% CI) | *P* value | |
| Group |  |  |  |  |  | |
| PFA | 0.712 (0.400, 1.266) | 0.247 |  | 0.791 (0.441, 1.417) | 0.430 | |
| CBA | 0.829 (0.465, 1.477) | 0.524 |  | 0.884 (0.496, 1.578) | 0.677 | |
| RFA | 1 [Reference] |  |  | 1 [Reference] |  | |
| Gender | 0.950 (0.616, 1.465) | 0.815 |  | 0.947 (0.607, 1.476) | 0.809 | |
| Age | 0.999 (0.975, 1.025) | 0.966 |  | 0.991 (0.965, 1.017) | 0.492 | |
| BMI | 0.917 (0.849, 1.091) | 0.730 |  | 0.939 (0.816, 1.068) | 0.657 | |
| LA diameter | 1.027 (0.982, 1.075) | 0.244 |  | 1.052 (1.002, 1.106) | 0.043 | |

PFA: Pulsed Field Ablation; CBA: Cryoballoon Aablation; RFA: Radiofrequency Ablation; BMI: Body Mass Index; LA: left atrium.

Figure S1. Study Flow Chart

**
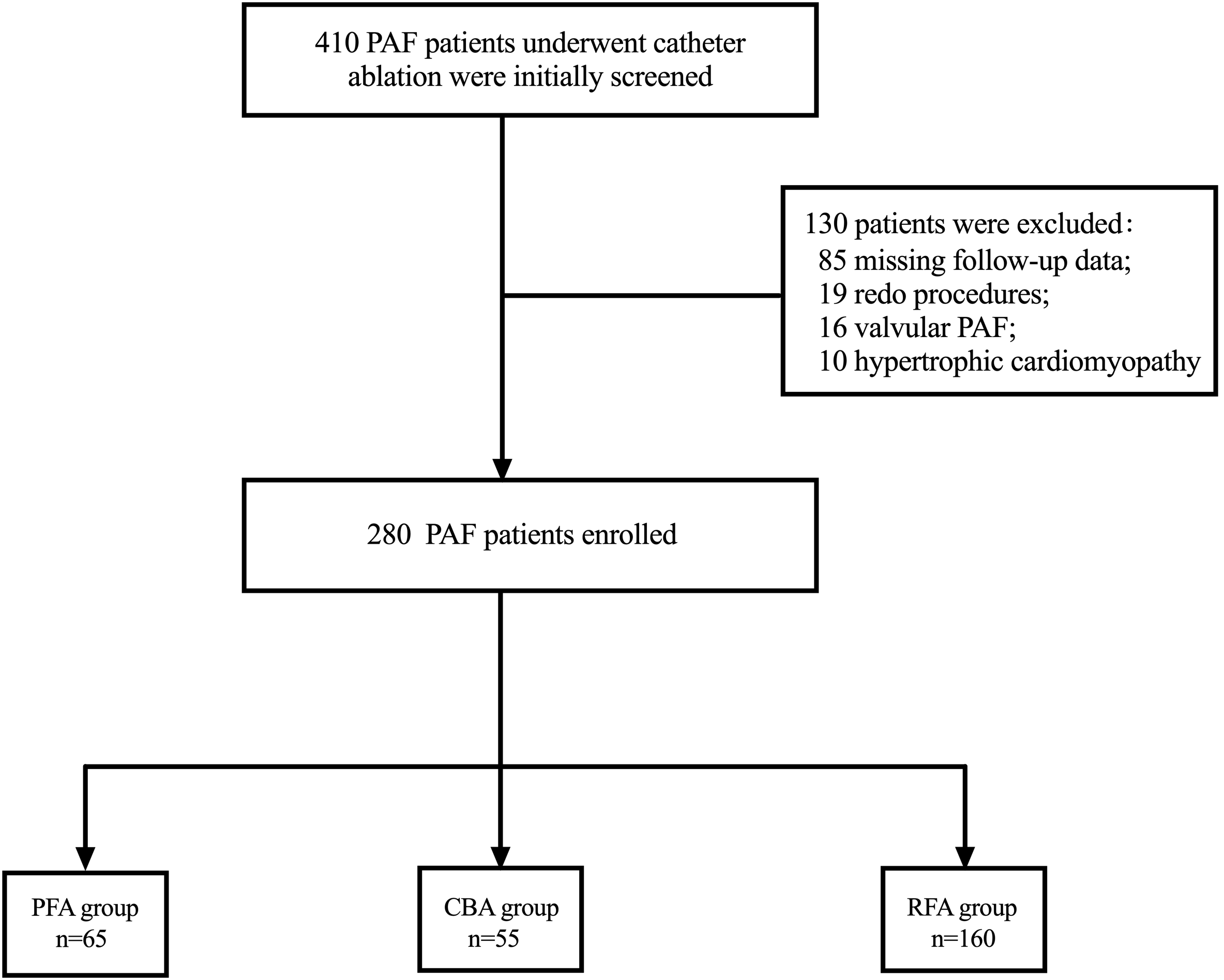
**

PAF: Paroxysmal atrial fibrillation; PFA: Pulsed Field Ablation; CBA: Cryoballoon Aablation; RFA: Radiofrequency Ablation.
